# The Nasopharynx Swab Test for Coronavirus Disease-2019 Is Mild and Will Not Cause Significant Pain and Anxiety: A Cross-Sectional Study Based on Psychiatrists

**DOI:** 10.3389/fcimb.2021.592092

**Published:** 2021-09-30

**Authors:** Wei Li, Han Zhou, Qian Guo, Guanjun Li

**Affiliations:** ^1^ Department of Geriatric Psychiatry, Shanghai Mental Health Center, Shanghai Jiao Tong University School of Medicine, Shanghai, China; ^2^ Alzheimer’s Disease and Related Disorders Center, Shanghai Jiao Tong University, Shanghai, China; ^3^ Department of Early Psychotic Disorder, Shanghai Mental Health Center, Shanghai Jiao Tong University School of Medicine, Shanghai, China

**Keywords:** COVID-19, women, anxious, nervous, nasopharyngeal swab test

## Abstract

**Background:**

Laboratory viral nucleic acid testing (NAT), such as the nasopharyngeal swab test, is now recommended as the gold standard for the diagnosis of Coronavirus disease-2019 (COVID-19). However, the nasopharyngeal swab testing process may cause some discomfort.

**Objective:**

To investigate the influence of nasopharyngeal swab tests on the anxiety and pain felt by psychiatric medical staff.

**Methods:**

A total of 174 psychiatric medical staff (namely 97 doctors, 68 nurses, and nine administrators) and 27 controls were included in the current study. A self-designed questionnaire was used to collect their general demographic information (age, gender, marriage, occupation, profession, smoking history, alcohol consumption history, tea drinking history, previous history of anxiety and depression) as well as their subjective experience, such as nausea, vomiting, coughing, worry, fear, etc, during nasopharyngeal swab collection. The Numerical Rating Scale (NRS) and the State-Trait Anxiety Inventory (STAI) were used to assess the subjects’ pain and state anxiety, respectively.

**Results:**

There were no statistical differences (p>0.05) in age, marriage, smoking history, a history of anxiety and depression, pain scores, and anxiety scores between different professions and genders. The results of partial correlation analysis (controlled for gender and history of depression or anxiety) indicated that the male gender was negatively correlated with being anxious (r=-0.148, p=0.037) and nervous (r=-0.171 p=0.016), although there was no significant difference in pain and anxiety between men and women. In addition, marriage might help women resist negative emotions.

**Conclusions:**

1) There will be mild discomfort during nucleic acid testing, but not enough to cause pain and anxiety; 2) women are more likely to be anxious and nervous during the nucleic acid testing.

## Introduction

Coronavirus disease-2019(COVID-19) has been spreading globally since the end of 2019. As of March 10, 2020, the global number of confirmed cases of COVID-19 has surpassed 118 000, and most cases (68.42%) occurred in China (Pan et al., 2020). To identify infected patients and begin clinical treatment in a timely manner, starting from January 15, 2020, the Chinese government issued seven successive versions of COVID-19 diagnostic and treatment guidelines. Laboratory viral nucleic acid testing (NAT), such as the nasopharyngeal swab test, is now recommended as the gold standard for the diagnosis of COVID-19 (Pan et al., 2020), and it has proven to be one of the most quickly established laboratory diagnosis methods in a novel viral pandemic, which can serve efficiently to confirm COVID-19 infection within 2 h (Liu et al., 2020).

Nasopharyngeal swab tests can be performed on several types of upper respiratory specimens, including washes, swabs, and aspirates (Frazee et al., 2018), however, it may cause some degree of discomfort, such as nausea and coughing, although they can be tolerated (Hansen et al., 2016). To our knowledge, there have been no studies exploring the severity of discomfort caused by nasopharyngeal swab tests and their associated factors. Therefore, we conducted this cross-sectional study to specifically examine the level of discomfort associated with the detection of COVID-19 by nasopharyngeal swabs among Chinese psychiatric medical staff.

## Materials and Methods

### Participants

This cross-sectional study was conducted with psychiatric medical staff from Shanghai mental health center between July 2 and 9, 2020. The inclusion criteria were as follows: 1) participants had taken a nucleic acid test within the past week; 2) nasal and pharyngeal swabs were tested simultaneously; 3) participants had to be Shanghai Mental Health Center staff, including doctors, nurses, and administrative staff; and 4) they were willing to be investigated. Exclusion criteria were as follows: 1) nucleic acid tests took more than a week; 2) non-psychiatric related major; 3) only nasal or pharyngeal swabs were performed; 4) the onset of anxiety and depression; or 5) participants refused to be investigated. Finally, 174 psychiatric medical staff working in Shanghai mental health center and 27 controls (such as family members or nursing workers of medical personnel) were enrolled in the study.

Ethical approval was issued by the Ethics Committee of Shanghai Mental Health Center, and all the participants had signed informed consent before the study was initiated.

### Investigation Tools

By using a self-designed questionnaire, we have obtained the general demographic information of the respondents, including their age, gender, marriage, occupation, profession, smoking history, alcohol consumption history, tea drinking history, previous history of anxiety and depression, as well as their subjective experience, such as nausea, vomiting, coughing, worry, fear, etc during nasopharyngeal swab collection.

### Psychopathology Batteries

The Numerical Rating Scale (NRS) and the State-Trait Anxiety Inventory (STAI) were used to assess the subjects’ pain and state anxiety, respectively. The numeral assessment scale represents the pain degree by 11 Numbers from 0 to 10, 0 means no pain, 10 means the most pain, and the subjects will select one of the Numbers according to his/her personal pain feeling, to represent his/her pain degree (Wikstrom et al., 2019). The NRS has become the most recommended scale as a result of patients’ preferences regardless of context and age (Hjermstad et al., 2011). The STAI was used to assess the participants’ state anxiety (i.e., feelings of anxiety at a given moment) (Wu et al., 2019). Each item is evaluated based on the severity of the symptoms (1 = not at all, 2 = some, 3 = moderate, 4 = very obvious). The STAI scores range from 20 to 80, with higher scores indicating more severe symptoms, and a score of 45.13 is considered as the cut-off value to determine whether the participants have anxiety (Abed et al., 2011).

### Investigation Method

In the current study, we used the Electronic “Questionnaire Star” as the surveying tool, and information was collected through WeChat friends circle forwarding. “Questionnaire Star” is a specialized online platform for questionnaire evaluation, voting, and other purposes. Compared with the traditional survey methods, “Questionnaire Star” has the obvious advantages of being a fast, low cost, and easy to learn, surveying tool (Li et al., 2019).

### Definition of Specific Variables

We used standardized questionnaires to collect the general demographic data of the respondents, such as their age, gender, profession, marital status, and feelings during nucleic acid testing, such as nausea, vomiting, coughing, and so on. All of the questions regarding the feelings during nucleic acid testing were answered as “yes” or “no”.

### Data Analysis

The continuous variables were expressed as the mean ± standard deviation, and the categorical variables were represented by frequency (%). The one-sample Kolmogorov-Smirnov test was utilized to explore whether the data were normally distributed. The chi-square test was used to compare the categorical variables, while the t-test and Mann-Whitney U-test were used to compare the continuous variables that did and did not have a normal distribution, respectively. Partial correlation analysis was used to assess the association between worry/fear and gender, and we had controlled for profession, smoking, and drinking tea. Correlation analysis was used to explore the relationship between NRS and STAI, and single factor ANOVA analysis was used to explore the impact of marriage on NRS and STAI scores in women. IBM SPSS Statistics for Windows, version 22.0 (IBM Corp., Armonk, NY, USA) was used for the statistical analysis. A p-value < 0.05 was considered significant.

## Results

### General Demographic Data of the Psychiatric Medical Staff

**Figure 1:**
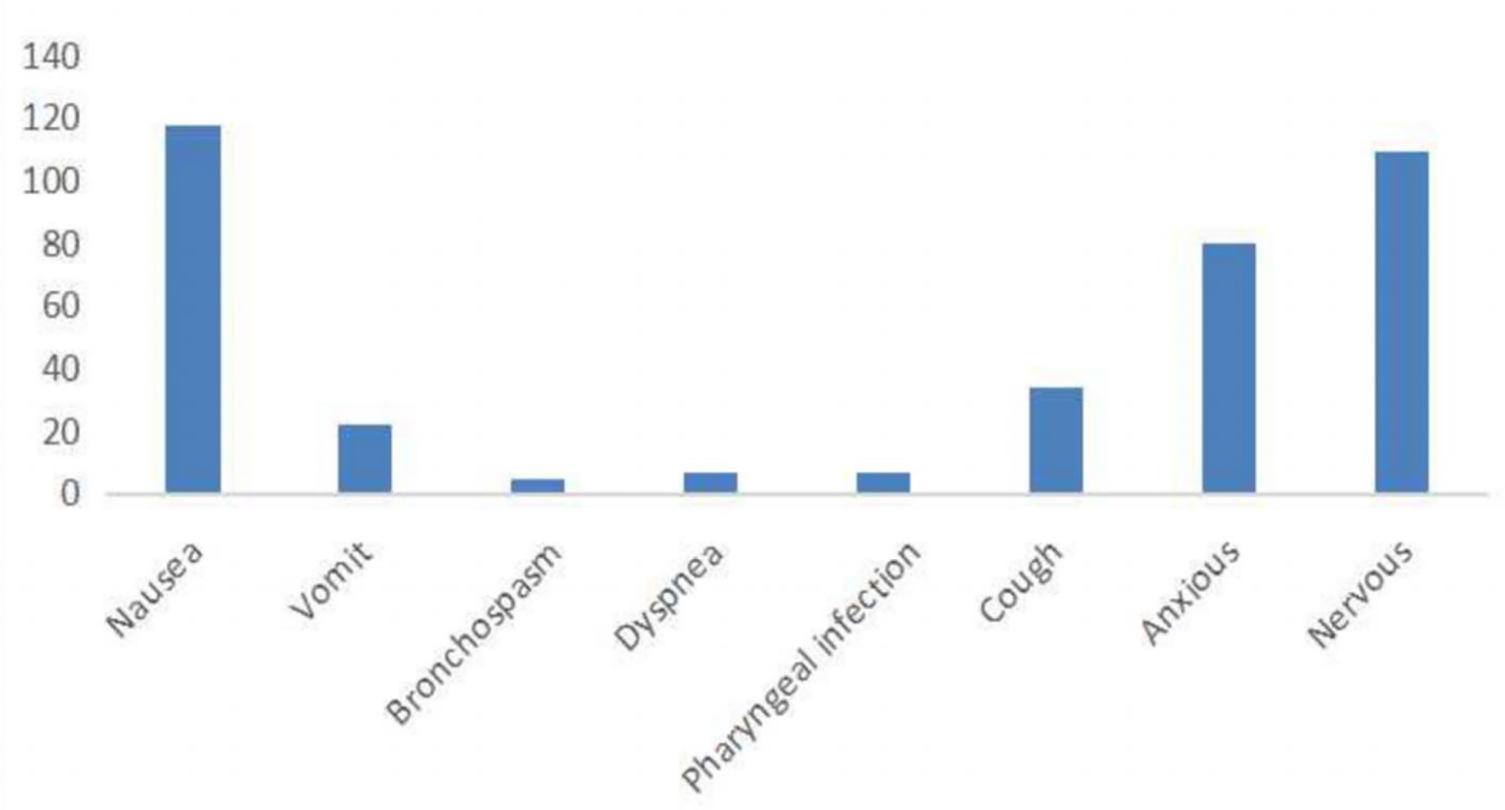
Common adverse reactions in nucleic acid testing.

We enrolled 201 participants in this study. Of them, 97 were doctors, which accounted for 48.3%, 68(33.8%) were nurses, nine (4.5%) were administrators, and 27(13.4%) were others. 118 (58.7%) felt nausea, 109(54.2%)felt nervous, 80(39.8%) felt anxious, 34(16.9%) coughed, 22 (10.9%) vomited, 5(2.5%) felt bronchospasm, 7(3.5%) felt dyspnea, and 7(3.5%) worried about pharyngeal infection. **Figure 1** presents the results. There were statistical differences (p<0.05) between professions, tea drinkers, alcohol drinkers, those who felt anxious, and those who felt nervous between the male group and the female group, while there were no statistical differences (p>0.05) in age, marriage, smoking history, a history of anxiety and depression, pain scores and anxiety scores. **Table 1** shows the results.

**Table 1:** General demographic information of the subjects.

Variables	Total (n = 201)	Male (n = 34)	Female (n = 167)	p
Marriage				
Married, n (%)	141 (70.1)	22 (64.7)	119 (71.3)	0.721
Not married,n (%)	56 (27.9)	11 (32.4)	45 (26.9)	
Divorced,n (%)	4 (2.0)	1 (2.9)	3 (1.8)	
Profession				
Doctors,n (%)	97 (48.3)	22 (64.7)	75 (44.9)	<0.001*
Nurses,n (%)	68 (33.8)	1 (2.9)	67 (40.1)	
Administrators,n (%)	9 (4.5)	3 (8.8)	6 (3.6)	
Others, n (%)	27 (13.4)	8 (23.5)	19 (11.4)	
Smoker				
Yes,n (%)	8 (4.0)	3 (8.8)	5 (3.0)	0.136
No,n (%)	193 (96.0)	31 (91.2)	162 (97)	
Alcohol drinker				
Yes,n (%)	23 (11.4)	14 (41.2)	9 (5.4)	<0.001*
No,n (%)	178 (88.6)	20 (58.8)	158 (94.6)	
Tea drinker				
Yes,n (%)	89 (44.3)	22 (64.7)	67 (40.1)	0.013*
No,n (%)	112 (55.7)	12 (35.3)	100 (59.9)	
A history of anxiety and depression				
Yes,n (%)	8 (4.0)	2 (5.9)	6 (3.6)	0.625
No,n (%)	193 (96.0)	32 (94.1)	161 (96.4)	
Feeling of nucleic acid detection				
Nausea,n (%)	118 (58.7)	20 (58.8)	98 (58.7)	1.000
Vomit,n (%)	22 (10.9)	2 (5.9)	20 (12.0)	0.382
Bronchospasm,n (%)	5 (2.5)	1 (2.9)	4 (2.4)	1.000
Dyspnea, n (%)	7 (3.5)	3 (8.8)	4 (2.4)	0.096
Pharyngeal infection, n (%)	7 (3.5)	1 (2.9)	6 (3.6)	1.000
Cough, n (%)	34 (16.9)	4 (11.8)	30 (18.0)	0.461
Feel anxious, n (%)	80 (39.8)	7 (20.6)	73 (43.7)	0.013*
Feel nervous, n (%)	109 (54.2)	10 (29.4)	99 (59.3)	0.002*
Anxiety based on State Anxiety Inventory				
Yes,n (%)	19 (9.5)	2 (5.9)	17 (10.2)	0.747
No,n (%)	182 (90.5)	32 (94.1)	150 (89.8)	
Age, y	34.58 ± 7.758	35.24 ± 8.818	34.44 ± 7.551	0.590
Pain scores	3.73 ± 2.074	3.82 ± 2.443	3.71 ± 1.998	0.765
Anxiety scores	31.32 ± 10.422	30.44 ± 12.524	31.50 ± 9.974	0.592

*p < 0.05.

### Comparison of Pain Scores and Anxiety Scores in Nucleic Acid Testing Between Medical Staff and Non-Medical Staff

Next, we classified 97 doctors, 68 nurses, and nine administrative staff into the medical staff group and the remaining 27 participants into the non-medical staff group, and compared the NRS and STAI scores between the two groups. Finally, we found no statistical difference (p>0.05) in NRS (3.80 ± 2.109 *vs* 3.22 ± 1.783) and STAI (31.57 ± 10.847 *vs* 29.70 ± 7.032) scores between the two groups, suggesting that there was no difference in the tolerance of medical personnel and non-medical personnel to nucleic acid testing.

### Relationship Between NRS Scale and STAI Scale

**Figure 2:**
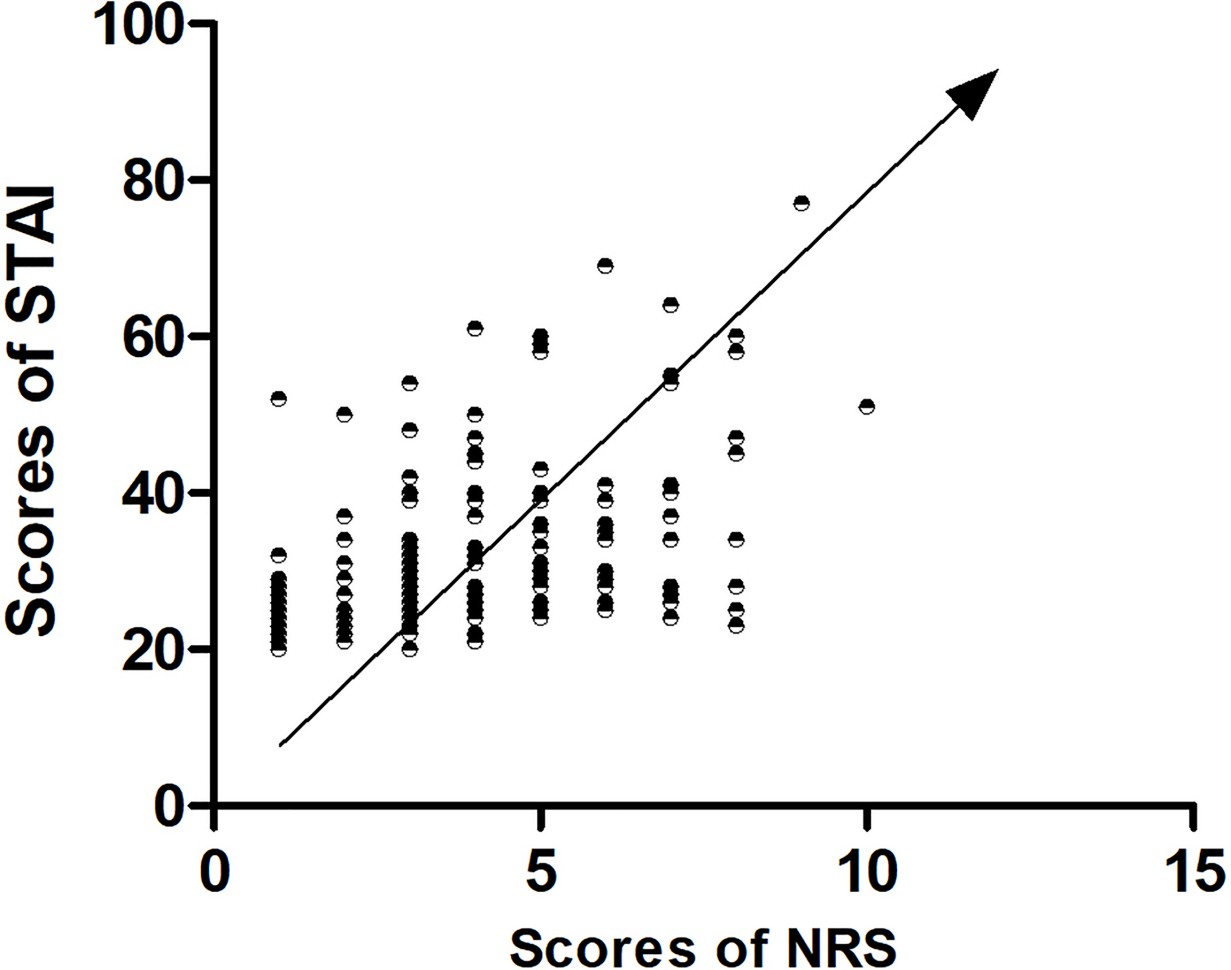
Correlation between NRS and STAI.

By using correlation analysis, we found that the total score of NRS was significantly correlated with the total score of STAI. **Figure 2** shows the results.

### The Relationship Between Gender and Anxious/and Nervous

The results of partial correlation analysis (controlled for profession, alcohol drinking, and tea drinking) indicated that the male gender was negatively correlated with feeling anxious (r=-0.148, p=0.037) and nervous (r=-0.171 p=0.016).

### The Effect of Marital Status on Women’s NRS Score and STAI Score

In order to explore the impact of marriage on women’s NRS score and STAI score, we then applied one-way ANOVA analysis LSD test, and finally found that married women scored less on NRS and STAI than unmarried women, while there was no statistical difference between the divorced group and the unmarried group, suggesting that marriage might help relieve women’s pain and anxiety. **Tables 2** and **3** present the results.

**Table 2:** The effect of different marital status on women’s NRS score and STAI score (ANOVA).

Variables	Married (n = 119)	Unmarried (n = 45)	Divorced (n = 3)	F	p
Total STAI score	30.62 ± 9.246	34.27 ± 11.525	24.67 ± 1.528	2.965	0.054
Total NRS score	3.48 ± 1.948	4.40 ± 1.970	2.33 ± 2.309	4.361	0.014*

*p < 0.05.

**Table 3:** The effect of different marital status on women’s NRS score and STAI score (LSD).

Variables	Marriage (i)	Marriage (ii)	Mean difference (i-ii)	S.E	p	95%confidence interval
Total STAI score	Married	Unmarried	-3.645	1.725	0.036*	-7.051~-0.238
		Divorced	5.955	5.763	0.303	-5.424~17.334
	Unmarried	Divorced	9.600	5.878	0.104	-2.007~21.207
Total NRS score	Married	Unmarried	-0.921	0.343	0.008*	-1.600~-0.240
		Divorced	1.146	1.145	0.319	-1.120~3.410
	Unmarried	Divorced	2.067	1.168	0.079	-0.24~4.37

*p < 0.05.

## Discussion

To my knowledge, this is the first study to explore the level of discomfort associated with the detection of COVID-19 by nasopharyngeal swabs among Chinese psychiatric medical staff, and we have got some interesting results: 1) there was mild discomfort during nucleic acid testing, but not enough to cause pain and anxiety; 2) there was no significant difference in discomfort between medical staff and non-medical staff during the process of nucleic acid testing; 3) women were more likely to be anxious and nervous during the nucleic acid testing; and 4) marriage might help relieve women’s pain and anxiety.

COVID-19 is associated with human-to-human transmission and has recently been found in the saliva of infected patients. Salivary diagnostics may provide an easy and cheap platform for early and quick diagnosis of COVID-19 (Sabino-Silva et al., 2020), so the oropharyngeal and nasopharyngeal (OP/NP) samples have been commonly used as a screening tool (Winichakoon et al., 2020). However, the process of taking a saliva sample can cause discomfort, such as nausea or bleeding, which may not be appropriate for all populations, especially those with thrombocytopenia (Sri Santosh et al., 2020). What’s more, it can also put health-care workers at risk of infection, so many people have expressed their nervousness and concern.

Because of the closed working environment in psychiatric hospitals, which are more prone to cluster infections, the Chinese government requires employees in every psychiatric hospital to undergo nucleic acid testing. In the current study, we investigated the pain and anxiety levels of psychiatric medical staff in Shanghai mental health center during nucleic acid testing (by nasopharyngeal swab) and found that the most common symptoms during nasopharyngeal swabs were nausea, nervousness, anxiety, coughing, and vomiting. However, these symptoms were mild and did not cause significant pain or anxiety. In addition, we investigated the emotional responses of medical staff and non-medical staff during the nucleic acid testing process, and we found no difference in pain and anxiety between the two groups, suggesting that the nucleic acid testing process did not cause too much pain and panic.

Next, we explored the factors that influence feeling anxious and nervous, and the results of partial correlation analysis (controlled for gender and history of depression or anxiety) indicated that women were positively correlated with feeling anxious and nervous, which was consistent with previous findings (Reisner et al., 2016; Howell and Weeks, 2017; Barbaro et al., 2018). However, we did not find that there was any effect of different occupations on feeling anxious and nervous, suggesting that this emotional response is universal, therefore, we should give more attention to women and do a good job in health education.

There are several mechanisms that might explain why women are more prone to negative emotions. First, women tend to show high anxiety and adopt negative ways to deal with negative emotions (Qi et al., 2020). Second, women are more likely to experience certain types of stressors, such as sexual trauma (Mayor, 2015). Third, higher negative emotions in women are associated with more severe mood disorders and are associated with depression, anxiety, and substance use disorders (Brady and Sinha, 2005). Fourth, compared with men, women reported greater sadness, anxiety, and physical feelings caused by stress when facing the same stress (Guinle and Sinha, 2020). What’s more, genes, hormones, and brain structure may also play a role in women’s moods (Gibson et al., 2011; Albert et al., 2015; Lamers et al., 2019; Robakis et al., 2019; Bower et al., 2020). Interestingly, we found that marriage helps women resist negative emotions, which was consistent with other studies (Kiecolt-Glaser and Newton, 2001; Boerner et al., 2014). We speculate that marriage provides women with security and emotional support and helps them cope with negative emotions in a positive way.

Finally, through correlation analysis, we found a positive correlation between the total score of NRS and the total score of STAI (**Figure 2**), suggesting that anxiety and pain are closely related. In fact, anxiety and pain often go hand in hand, and it is hard to pinpoint their cause and effect. Similarly, since our current study was only a cross-sectional study, we could not continue to analyze the internal relationship between the two factors, which was a limitation of our current study.

We have to admit that our study has certain limitations: first, it was just a cross-sectional study that could not establish a causal link between gender and emotional response; second, our sample size was relatively small, which reduces the reliability of the study.

## Conclusions

The discomfort of COVID-19 detected by nasopharynx swab is mild, and will not cause obvious pain and anxiety, however, it is still necessary to pay attention to the adverse emotional reactions of women.

## Data Availability Statement

The raw data supporting the conclusions of this article will be made available by the authors, without undue reservation.

## Ethics Statement

The studies involving human participants were reviewed and approved by the Ethics Committee of Ethics Committee of Shanghai Mental Health Center. The patients/participants provided their written informed consent to participate in this study.

## Author Contributions

WL and GL contributed to the study concept and design. QG and HZ collected the data. WL analyzed the data and drafted the manuscript. All authors contributed to the article and approved the submitted version.

## Funding

This work was supported by grants from the Clinical research center project of Shanghai Mental Health Center (CRC2017ZD02), the Cultivation of Multidisciplinary Interdisciplinary Project in Shanghai Jiaotong University (YG2019QNA10), and the curriculum reform of Medical College of Shanghai Jiaotong University, the Feixiang Program of Shanghai Mental Health Center(2020-FX-03).

## Conflict of Interest

The authors declare that the research was conducted in the absence of any commercial or financial relationships that could be construed as a potential conflict of interest.

## Publisher’s Note

All claims expressed in this article are solely those of the authors and do not necessarily represent those of their affiliated organizations, or those of the publisher, the editors and the reviewers. Any product that may be evaluated in this article, or claim that may be made by its manufacturer, is not guaranteed or endorsed by the publisher.
